# Efficient generation of connectivity in neuronal networks from simulator-independent descriptions

**DOI:** 10.3389/fninf.2014.00043

**Published:** 2014-04-22

**Authors:** Mikael Djurfeldt, Andrew P. Davison, Jochen M. Eppler

**Affiliations:** ^1^PDC Center for High-Performance Computing, KTH Royal Institute of TechnologyStockholm, Sweden; ^2^International Neuroinformatics Coordinating FacilityStockholm, Sweden; ^3^CNRS, Unité de Neurosciences, Information et ComplexitéGif sur Yvette, France; ^4^Institute of Neuroscience and Medicine (INM-6) and Institute for Advanced Simulation (IAS-6), Jülich Research Centre and JARAJülich, Germany

**Keywords:** model description, connectivity, neural simulation, CSA, NEST, PyNN, Python, large-scale modeling

## Abstract

Simulator-independent descriptions of connectivity in neuronal networks promise greater ease of model sharing, improved reproducibility of simulation results, and reduced programming effort for computational neuroscientists. However, until now, enabling the use of such descriptions in a given simulator in a computationally efficient way has entailed considerable work for simulator developers, which must be repeated for each new connectivity-generating library that is developed. We have developed a generic connection generator interface that provides a standard way to connect a connectivity-generating library to a simulator, such that one library can easily be replaced by another, according to the modeler's needs. We have used the connection generator interface to connect C++ and Python implementations of the previously described connection-set algebra to the NEST simulator. We also demonstrate how the simulator-independent modeling framework PyNN can transparently take advantage of this, passing a connection description through to the simulator layer for rapid processing in C++ where a simulator supports the connection generator interface and falling-back to slower iteration in Python otherwise. A set of benchmarks demonstrates the good performance of the interface.

## 1. Introduction

The central nervous systems of vertebrates and many invertebrates have complex patterns of connections. In developing neuronal network models of such systems, there are two main tasks related to connection patterns. The first is to express the connectivity in a machine-readable format, e.g., in code or in a configuration file. The second is to explain the connectivity unambiguously in prose in an article or book, so that the model can be understood and reproduced by someone else (Nordlie et al., 2009).

For expressing connectivity in code, three methods are commonly used: (1) procedural code written by the modeler, using low-level operations such as connecting pairs of neurons or connecting a single neuron to a group; (2) a library of pre-defined, parameterized connection routines; (3) an explicit list of connections. Each of these have their limitations. Procedural code and libraries limit the connectivity descriptions to a single language and often a single simulator, making it hard to port models from one simulator to another. Procedural code takes time to write, and will have more bugs than a library, since library code is likely to be more thoroughly tested and reused. On the other hand, the connectivity patterns available from a library are inevitably more limited than those that can be achieved with user-written code. Using an explicit list of connections is largely independent of a particular simulator or programming language, but may cause problems of storage and input/output efficiency, and does not enable conceptual descriptions of connectivity. All of these methods make it difficult to explain the connectivity in a scientific text. For a fuller discussion of these issues see Crook et al. (2012).

These problems can be reduced or avoided by using a general purpose connectivity-generating software library such as the connection-set algebra (CSA; Djurfeldt, 2012) or the NineML graph library (Raikov and De Schutter, 2010). Such libraries allow simulator-independent specification of connectivity and enable high-level, declarative descriptions which do not constrain how the connectivity should be realized in code and hence give scope for optimization and parallelization within the simulator software. The CSA in addition supports succinct, unambiguous descriptions of connectivity in text, using a mathematical notation.

The adoption of new, simulator-independent methods for expressing connectivity, such as CSA, is hindered by the effort needed to add support for a given connectivity-generating library to a simulator. This effort must, in general, be repeated for each simulator that aims to support the library. The exception to this is if using a simulator-independent modeling interface such as PyNN (http://www.neuralensemble.org/PyNN; Davison et al., 2009), which supports multiple simulators, and could also support multiple connectivity-generating libraries without the need to support it in each simulator. PyNN, however, is written in Python, which means that processing the CSA description will be slower than if using C/C++ (the languages in which many simulators are written). Furthermore, whether using a simulator-independent interface or not, a new programming effort is needed if another new method for expressing connectivity is developed.

To minimize the effort required of simulator software developers, and allow modelers to flexibly choose among simulators and libraries which generate connectivity, we have developed a generic *connection generator* interface, which enables the use of CSA and similar libraries in different neuronal simulators. The interface makes both the simulator and the connectivity-generating library replaceable and therefore gives maximum flexibility to the modeler.

In this article we first describe the connection generator interface. As an example of a connectivity-generating library, we then give a brief overview of CSA and of different software libraries that support it using CSA in Python and C++ code. We show how the connection generator interface has been integrated in the NEST simulator (http://www.nest-simulator.org; Gewaltig and Diesmann, 2007), enabling the use of connection libraries such as CSA, before describing its use from higher software layers, such as the PyNN framework. Finally, we provide benchmarks for different use case scenarios of the interface.

## 2. The connection generator interface

To allow users to flexibly choose among simulators and libraries which generate connectivity, we have developed an interface that abstracts the simulator and the connectivity-generating library from each other, making both the simulator and the connectivity description library replaceable.

**Figure 1:**
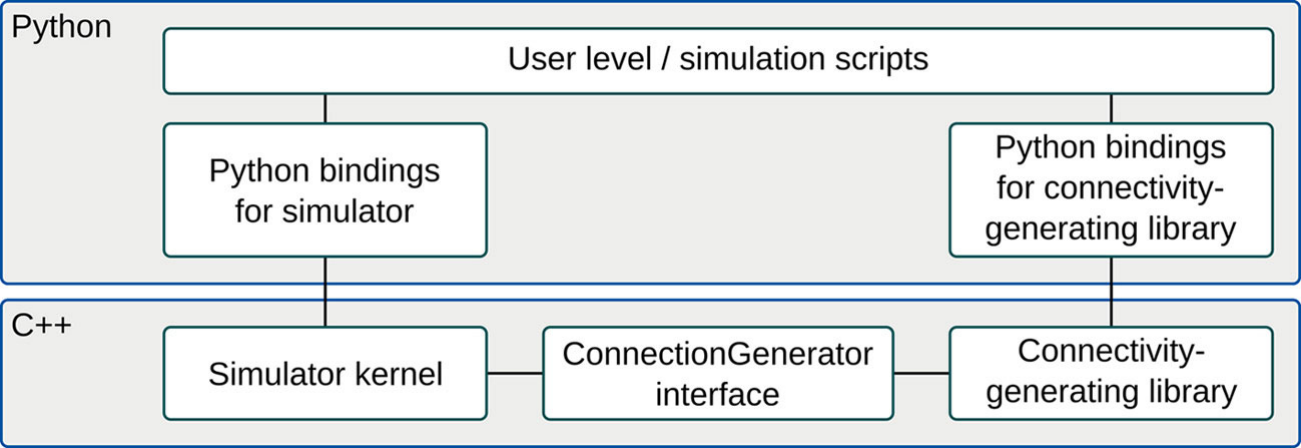
**Block diagram of the connection generator interface and the components involved in a typical usage scenario**. The central component is the ConnectionGenerator class itself. It can connect to different simulators (e.g., NEST) and to different connectivity-generating libraries (e.g., CSA). In this example, Python is used as scripting language for both simulator and connectivity- generating library.

A connectivity-generating library, such as csa or libcsa of section 3.3, is used to create an object, a *connection generator*, representing network connectivity. In the case of a CSA library, this object is a connection-set, but it could also be a graph constructed from graph primitives. The connection generator interface provides a C++ level interface to such objects which allows software external to the connectivity-generating library to efficiently iterate through connections represented by the object. In the example shown in Figure 1, the connection generator interface is used to combine a Python-scripted simulator with a Python-scripted connectivity-generating library. A connectivity generating object is assembled at the Python level. If libcsa is used, the resulting object will be a C++ object with a Python wrapper (see lower two boxes to the right in the figure). The object is used by a C++ simulator kernel (lower box to the left) to specify network connectivity. By providing connectivity-generating software that implements the connection generator interface as dynamically linked libraries, multiple such libraries can be loaded into the Python runtime environment and used simultaneously without a need to recompile the simulator.

### 2.1. The interface

In neuronal network simulations we often want to specify a “projection” between a source population of neurons and a target population. A projection consists of many individual connections between the populations. In nature, one such connection corresponds to an axon forming a synaptic contact on the spine/dendrite of a receiving neuron. A ConnectionGenerator object is modeled on the concept of a projection. The neurons in the source and target populations are enumerated using consecutive, non-negative integer indices starting from 0. These indices are used to specify source and target neuron identity for a connection and constitute an abstraction barrier between the actual elements of a population and the connection generator. This allows the elements to be either neurons (appropriate for networks of point neurons) or individual synapses (appropriate for networks with morphologically-detailed neurons).

The principle of operation of the connection generator interface is an iteration over connections represented by the object. A simulator, or other software using a connection generator, repeatedly calls a function next() until Boolean false is returned. Connections are represented by source and target index, together with zero or more connection parameters. The number of parameters is called the *arity* of the ConnectionGenerator.

Connection generators can be flexible with regard to the sizes of the source and target populations. The source and target index sets that should be iterated over for the populations at hand are each specified through the interface as a *mask*. In the case of a parallel simulator, a specific mask is given per MPI rank. Typically, the source index set is the same in all such masks while the target index sets are unique for each rank and non-overlapping. Each rank should provide all masks (i.e., the set of masks for all ranks). Some connection generators can use such information to avoid the need for communication with other ranks.

The interface is designed as an abstract base class in C++ and consists of the following virtual functions:

int arity(): Return the number of per-connection values associated with this generator. Values can be parameters like weight, delay, time constants, or others.
int size(): Return the number of connections represented by this generator.
void setMask (Mask& mask): Inform the generator about which source and target indices are available. A Mask represents the available nodes in the network for which to create connections.
void setMask(std::vector<Mask>& masks, int local): This version of setMask is used by parallel simulators and informs the connection generator on the local rank about the masks of all ranks. Different ranks usually have different masks since they are responsible for different subsets of the connections of the network. Some connection generators can use such information to avoid the need for communication with other ranks.
void start(): Start an iteration. This function must be called before the first call to next().
bool next(int& source, int& target, double* value): Advance to the next connection or return false, if no more connections are available from the ConnectionGenerator. Source and target indices of the connection as well as associated parameters are written into source, target and the array pointed to by value, respectively. The order of iteration is according to increasing index, with all sources iterated over per target.

### 2.2. Libneurosim

If the C++ header file for the connection generator interface definition and its supporting code were placed in the simulator or connectivity-generating library source trees, it would be duplicated over simulators or libraries. As new versions of the interface are developed, the situation would quickly become unmanageable. The connection generator interface has the desirable property that it is a symmetric abstraction barrier: it both allows a given simulator to use any library supporting the API *and* allows the same library to be used from any simulator supporting the API.

To create a space in which to put interfaces and other code of generic use for neuronal network simulation software, we have developed the neurosim library (http://software.incf.org/software/libneurosim). More precisely, *libneurosim* is a software package which currently consists of two main component libraries: libneurosim, providing C++ level support code and libpyneurosim, providing Python support code.

libneurosim provides the ConnectionGenerator interface (Figure 1, bottom). It also contains a registry for XML parser functions. Different types of connection generators can be described by different XML-based languages. For example, the csa library can serialize connection-set expressions using a MathML-based language. A connectivity-generating library may provide a parser for one or more such languages. Conversely, the same XML-based language might be used by one or more connectivity-generating libraries. The XML parser registry maps XML tags identifying specific languages to specific connection libraries. The interface to the registry consists of three static methods in the connection generator interface:

void selectCGImplementation (std::string tag, std::string library): Associate the parent node tag tag with the library library. The library named library will be dynamically loaded and will invoke the libneurosim function registerConnection GeneratorLibrary to register its XML parser.
ConnectionGenerator* fromXML (std::string xml): Parse the XML representation xml of a connection generator and return it. The function dispatches to parsers of different libraries depending on previous calls to selectCGImplementation.
ConnectionGenerator* fromXMLFile (std:: string fname): Same as previous function, but read the XML stream from the file with pathname fname.

libpyneurosim currently contains generic support for registering new Python connection generator types and unwrapping instances of such objects:

void registerConnectionGeneratorType (Check FuncT, UnpackFuncT): Register a new type checking and unwrapping function.
isConnectionGenerator (PyObject* pObj): Check if pObj is a known connection generator type (as identified by previously registered checking functions).
ConnectionGenerator* unpackConnectionGenerator (PyObject* pObj): Unwrap the connection generator in pObj and return it.

## 3. The connection-set algebra

The connection-set algebra^1^ (CSA) is a declarative formalism for the specification of network connectivity which can be used both when describing a network to a fellow researcher and when implementing a model for a simulator. CSA expressions define connection-sets. A connection-set is the set of edges in a network graph, together with parameters associated with those edges. The abstract nature of CSA creates a clean separation between CSA and other aspects of model or simulator infrastructure. CSA expressions consist of pre-defined elementary connection-sets and operators such that new connection-sets can be defined in terms of existing ones. It enables a succinct and precise description and definition of connectivity in terms of such expressions. By allowing connection-sets to be infinite, they can represent connectivity patterns of arbitrary size in addition to connectivity of specific networks. There are ways to implement CSA on a computer which are both efficient and scalable on a parallel computer. The parallelization is transparent to the user since intersection operators in the algebra can efficiently subdivide a connection-set among processes.

CSA is currently focused on the description of synthetic connectivity as opposed to connectivity obtained through data acquisition, but it can also be used when all network connections are given explicitly or in cases where only some elements of the connectivity are explicitly specified.

### 3.1. Connection-sets

To give a flavor of CSA, we will now further introduce some of its concepts. Given source and target neuron (or other object) populations *P_s_* and *P_t_* of fixed, finite size, a CSA connection-set is defined as the set of connections between *P_s_* and *P_t_* along with zero or more per-connection parameters such as weight or delay. As for connection generators, elements of populations are enumerated using non-negative integers. Thus, a population *P* is enumerated and represented by an index set 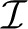. One connection from *P_s_* to *P_t_* is represented by the pair (*i, j*), where *i* ∈ 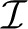
_*s*_, *j* ∈ 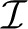
_*t*_. Such indices are similar to the GIDs (Global IDentifiers) of the NEURON and NEST simulators but normally start at 0 and run consecutively for each population. Introducing this abstraction, in the form of a mapping from objects in the simulation domain to integers, gives at least two major advantages: 1. The CSA need not be aware of the nature of the objects in *P*—they could just as well be synaptic boutons as neurons. 2. The index set 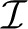 can be chosen to be infinite. This is useful when generically specifying a connectivity pattern independent of the sizes of the specific source and target populations for which it will later be used. This is further discussed in section 3.2.

**Figure 2:**
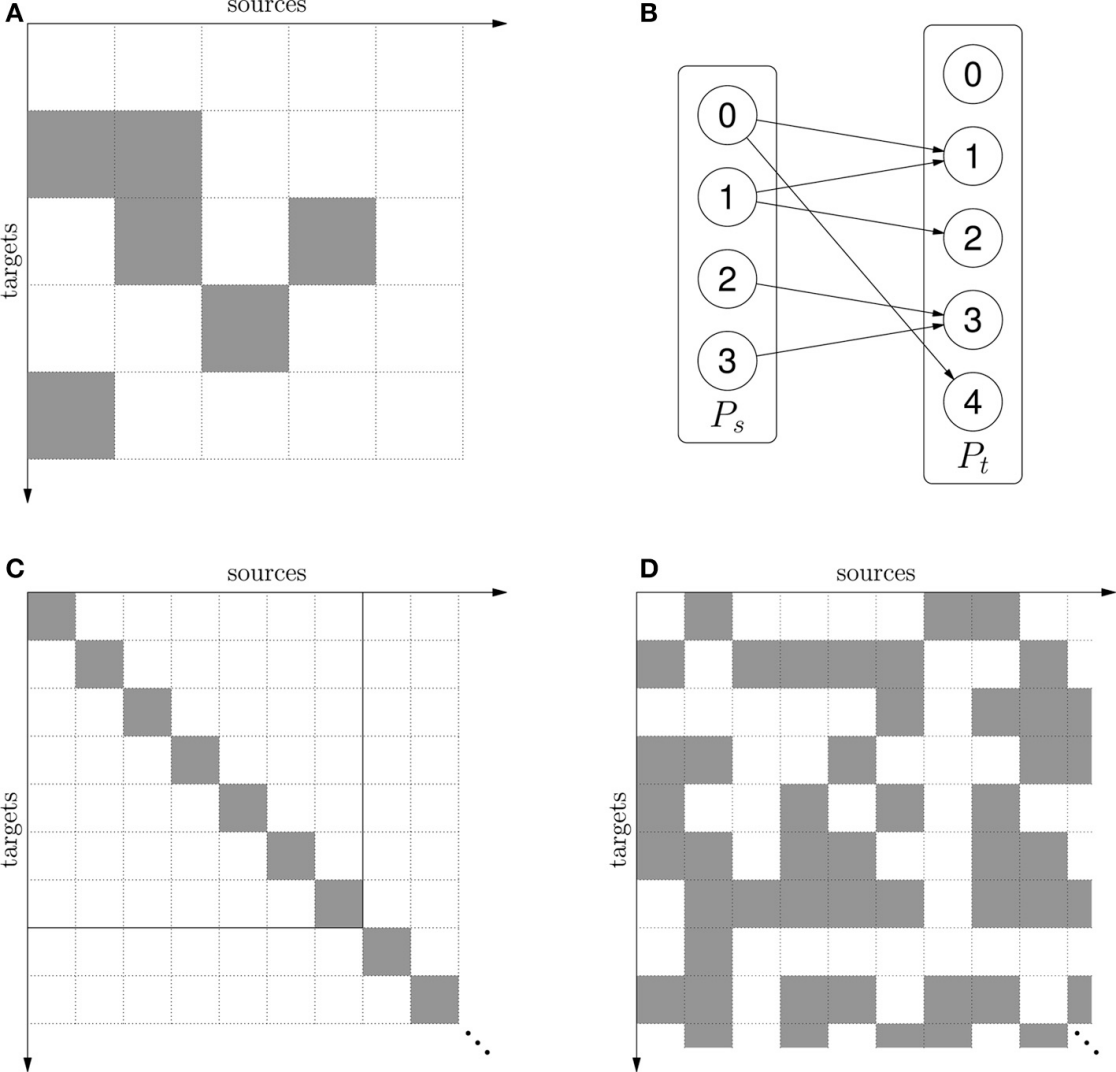
**(A)** The mask *M* = {(0,1), (1,1), (1,2), (3,2), (2,3), (0,4)} shown as a connection matrix. Gray squares represent existing connections. **(B)** Network connectivity when the mask in **(A)** is applied to source population *P_s_* and target population *P_t_*. **(C)** The one-to-one mask δ. The mask is infinite, but finite portions can be cut out when applied to finite source and target populations. This is illustrated by the solid square for source and target size 7. When source and target population is the same, δ represents self-connections. **(D)** The mask ρ(0.5) − δ = random connectivity without self-connections.

A connection-set with zero parameters is called a *mask*. A mask states which connections exist. In the example of the mask^2^ *M* = {(0,1), (1,1), (1,2), (3,2), (2,3), (0,4)} it can be regarded either as a connection matrix (see Figure 2A) or as a Boolean indicator function:

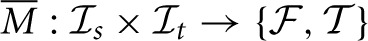



In the example *M*(0,0) = 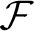 while *M*(1,1) = 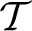 (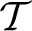 stands for *true*, 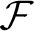 for *false*). If the mask is combined with a source population *P_s_* and a target population *P_t_*, the result is the network (*P_s_*, *P_t_*, *M*) shown in Figure 2B.

In CSA, connections can be parameterized through functions mapping connections to values:

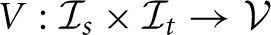



where 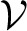 is some codomain, e.g., real numbers. In Djurfeldt (2012), such a function is called a *value set*. An example of a value set is distance dependent delays with added noise drawn from a clipped random normal distribution. A value set is typically used to assign a weight or delay to connections.

A CSA *connection-set*, *C*, is a tuple of a mask and zero or more value sets:

$$C=(\bar{M},V_{0},V_{1},\ldots )$$

The number of value sets of a connection-set, i.e., the number of values associated with each connection, is called its *arity*. A connection-set with arity 0, (*M*), is for all purposes equivalent to a mask, *M*, and the two can be used interchangeably.

### 3.2. An algebra for connectivity structure

In CSA, the concepts defined in the previous section have been developed into a formalism for describing connectivity structure. Assume that a mask specifies connectivity for a single population such that *P_s_* = *P_t_* = *P* and that we are interested in describing self-connections, i.e., every neuron in *P* should be connected to itself. If the size of *P* is 4, the mask {(0, 0), (1, 1), (2, 2), (3, 3)} could be used. If the size is 2, the mask {(0, 0), (1, 1)} would be appropriate. Such masks contain not only information about connectivity structure but also about population size. We can generalize by stripping off the population size information and allowing the index set 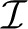 and mask to be infinite, defining the one-to-one mask δ:

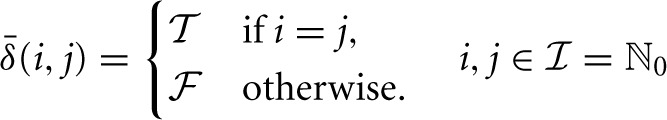



Now δ only encapsulates the concept of self- or one-to-one connectivity structure (depending on whether the source and target populations are the same or different) without reference to size, i.e., we can describe a connectivity pattern independently of any specific network. Finite portions can be cut out of such infinite connection-sets when applied to finite populations (see Figure 2C).

CSA provides a set of elementary connection-sets such as δ. Another example is the random mask ρ(*p*, a parameterized connection-set which captures the concept of Erdős-R’enyi connectivity. ρ(*p*) can be regarded an infinite matrix of independent, Bernouilli distributed, random variables with parameter *p*, such that their realizations form an infinite mask. Thus, for *R* = ρ (*p*) (where the interpretation is that *R* is the mask formed from realizations)

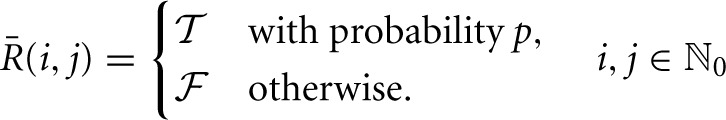



By using CSA *operators* such as intersection (∩), union (∪), and set difference (−), connection-sets can be combined into expressions. For example, the idea of “random Erdős-R’enyi connectivity without self-connections” can be represented by the CSA expression ρ(*p*) − δ (see Figure 2D). A mask representing all possible connections between two finite populations can be formed by taking the Cartesian product of their index sets, 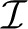
_*s*_ × 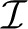
_*t*_. Intersecting with this mask can turn an infinite connection-set, representing connectivity structure, into a connection matrix between the populations. For example, the finite part of the matrix in Figure 2C is {0, …, 6} × {0, …, 6} ∩ δ. For a more in-depth description of the CSA and principles of implementation see Djurfeldt (2012).

### 3.3. Implementations

There currently exist three implementations of CSA. They internally represent connection-sets as iterators and CSA expressions as trees of such iterators. The original implementation is written in C++ and is part of the SPLIT simulation library (Djurfeldt et al., 2005). It was used to specify the connectivity of the KTH cortex model (Djurfeldt et al., 2008)—a model with three hierarchical levels of structure. The second implementation is written in Python and is available as the Python library csa. Here, the aims were 1. to get an easily usable and extensible demonstration of CSA and 2. to experiment with new ways to implement CSA. This implementation has been released as free software under the GPL and is available at the INCF software center (http://software.incf.org/software/csa). A third implementation in C++, libcsa, is currently under development and will also be released under the GPL. It provides Python bindings such that CSA objects can be formed by Python level expressions with similar syntax as used with the csa library. For further information about this syntax, the reader is referred to the tutorial in the csa package. The benchmarks in this article (section 6) were performed using the latter two implementations in Python and C++, csa and libcsa.

## 4. Using connection generators in nest

NEST is a simulator for large networks of point neurons or neuron models with few electrical compartments (http://www.nest-simulator.org; Gewaltig and Diesmann, 2007). It is suited for a broad range of neuronal network modeling approaches and runs on a large variety of computer architectures. NEST is parallelized using OpenMP (OpenMP Architecture Review Board, 2008) and MPI (Message Passing Interface Forum, 1994) and scales well on ordinary desktop computers to large clusters of multi-core processors and supercomputers (Helias et al., 2012).

The network description is a script, written either in SLI, NEST's built-in simulation language, or in Python, using the Python interface to NEST (PyNEST; Eppler et al., 2009). To build a network in NEST, the user first creates the neurons of the network and devices for stimulation and measurement using the Create function and then connects the elements with each other.

### 4.1. Native connection functions

The most basic way to set up connections is using the Connect function, which takes a list of pre-synaptic neurons (or devices) and a list of the same amount of post-synaptic neurons (or devices) and connects the corresponding elements in a one-to-one fashion. Because of the function call overhead, this function is not very efficient to use when creating large networks.

To avoid such overhead, the functions Convergent Connect and DivergentConnect can be used to create multiple connections with a single call. In addition, randomized variants for both of these functions exist to support the user in creating networks on the basis of knowledge of connectivity statistics. However, random connection parameters (e.g., weight, delay, or time constants) need to be specified by user code and supplied to NEST after the creation of connections.

### 4.2. Topology module

To ease the creation of complex networks with spatial structure, NEST provides the Topology Module (Plesser and Enger, 2013). It supports the user in connecting neurons and initializing synapse parameters based on their topological relationships in the network. In contrast to the CSA, the topology module is very much tailored to building structured networks consisting of layers in NEST with minimal overhead. The CSA has a wider focus in that it is simulator-independent and supports arbitrary connectivity patterns that can also include repetitive elements. We are currently investigating if future versions of the Topology Module can be based on the CSA.

### 4.3. Supporting connectivity-generating libraries

As detailed above, NEST provides multiple methods for connecting neurons into a network. However, while the native routines scale very well (Helias et al., 2012), they are only suitable for creating simple patterns such as convergent/divergent connectivity without looping over them in user code. On the other hand, the topology module (Plesser and Enger, 2013) allows the creation of more complex structures, but requires neurons to be organized in special data structures (e.g., layers).

To support the connection generator interface in NEST and thus make more connectivity-generating libraries available to users, we created the ConnectionGeneratorModule. It is implemented as a plugin for NEST which extends both user interfaces, SLI and PyNEST, and builds on libneurosim (see section 2.2).

All neurons and devices in NEST are identified uniquely by an integer number, their global id (*GID*). As all existing connection routines in NEST work either on single GIDs or on lists of GIDs, we decided to also use this convention when a user specifies cells for a connection generator. These GIDs are internally mapped to contiguous ranges of integer indices starting at zero, for use by the connection generator. Our new interface for using connection generators in NEST consists of the following functions:

CGConnect takes a ConnectionGenerator *cg*, lists of GIDs for *pre*- and *post*-synaptic populations, and a *param_map*. It creates the connections between neurons in *pre* and *post* as prescribed by the rules in *cg*. The parameter map *param_map* maps parameter names (e.g., weight, delay) to their index for the parameter value vector created by the call to next() in the connection generator interface (see section 2.1). In the current implementation, only arities 0 and 2 are supported.
CGParse takes a serialized version of a connection generator in the string *xml* and returns the corresponding ConnectionGenerator object. A special use of this function exists on supercomputers, where Python is often not available on the compute nodes, or where the memory and performance penalty would not be acceptable and a pure SLI-based solution is preferable.
CGParseFile takes a file name *fname* and parses the serialized version of a connection generator contained therein.
CGSelectImplementation takes an XML tag *tag* representing the parent node of a serialized connection generator and the name of a library *library* to provide a parser for such an XML file. This information determines which library should carry out the parsing for CGParse and CGParseFile.

In order to use the new interface in NEST, the user first has to construct a ConnectionGenerator object. This can be done at the Python level by either using csa or the Python bindings of libcsa (see section 3.3). When PyNEST is used, this object can be directly given to CGConnect, which wraps the ConnectionGenerator object into a SLI Datum of type connectiongeneratortype that can be handed over to NEST's simulation kernel. It is then iterated at the C++ level in case of libcsa, or by calling back into Python in case of csa.

Another way to construct a ConnectionGenerator object is by parsing an XML serialization of the object. Such a serialization could be created at the Python level, created by an external tool, or written by hand. At the SLI level this serialization can then be given to one of the SLI functions CGParse or CGParseFile, which reinstantiate the original object using the functions fromXML or fromXMLFile in the connection generator API. This object (of type connectiongeneratortype) can be given to SLI's version of the CGConnect function. Note that the step of creating the serialization of the ConnectionGenerator can also be carried out on another machine. In this way, simulations using CSA can be run on machines where Python is not available.

**Figure 3:**
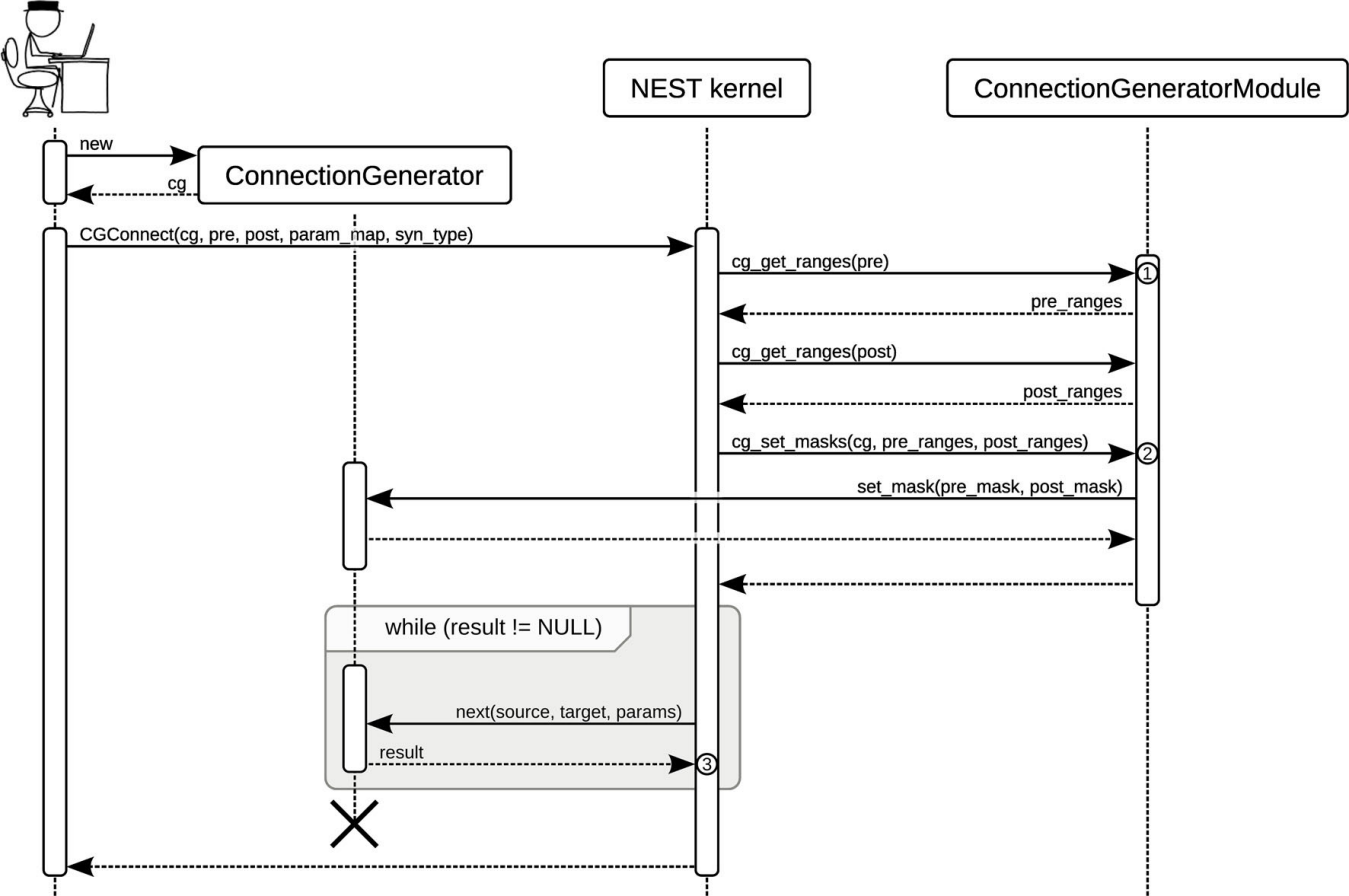
**Sequence diagram showing the function calls during the use of the connection generator interface in NEST**. The user first creates a ConnectionGenerator object *cg*. She then calls PyNEST's CGConnect() function. ① The function cg_get_ranges() returns all contiguous ranges of global IDs (GIDs) in the given list as a vector of closed intervals, still using the GID representation. ② At this point in time, the ConnectionGenerator Module needs to translate the GIDs to connection generator indices, which run from 0 and enumerate the elements of the given *pre*- and *post*-synaptic populations. The result of the translation (*pre_mask* and *post_mask*) is used to set the masks on *cg*. ③ The NEST kernel iterates the connection generator by calling next() until there are no more connections. For each received connection, it creates the connection by calling Network::connect(). If a non-empty *param_map* was given to CGConnect, the connection's weight and delay are taken from the value-set in *cg*.

Figure 3 shows the different entities in NEST involved in a user call to CGConnect in PyNEST. After setting the masks for the connection generator to tell it which neurons are local and which are remote (see section 2.1), the NEST kernel iteratively calls next(). This function returns source and target indices, and values for weight and delay if the arity of the connection generator is 2, until there are no more connections. The connections are internally established one by one calling NEST's basic Network::connect() function at the C++ level.

## 5. Using connection generators in PyNN

PyNN (http://www.neuralensemble.org/PyNN; Davison et al., 2009) is a simulator-independent API for describing neuronal networks in Python. Given a PyNN/Python model description, the user can choose which simulator to use without needing to change the model script. This is achieved through a set of *simulator backends*. Each backend is a Python module that implements the API for a specific simulator, for example by providing a mapping from standard model names and units in PyNN to simulator-specific ones.

In PyNN, a neuronal network is built from Populations of neurons and Projections between them. Each Projection is created by a Connector, which knows how to set up the individual connections. Different Connectors exist and allow to set up a variety of different deterministic and probabilistic connection patterns.

To enable them to be simulator-independent, each Connector is implemented in terms of lower-level commands, typically one-to-one or convergent connect functions, available in all simulators. However, because these commands are low-level this approach entails a certain performance penalty due to transfer of source/target lists and connection parameters and function call overhead.

To overcome the problem of excessive data transfers between PyNN and the simulator, a backend can supply customized Connector implementations that are more efficient than using the default, simulator-independent implementations. Such custom implementations can be iterated and expanded at the simulator level and thus avoid overhead. Moreover, they allow efficient parallelization at the lower software layers. Prior to the creation of the connection generator interface, however, the limitation of this was that a separate custom implementation had to be written for each PyNN Connector.

Since version 0.7, PyNN has provided the generic CSAConnector, which can iterate a CSA connection-set and issue one by one connect calls to the different simulators through their backends. Internally, the Connector executes the following two steps:

1. Form a finite connection-set adapted to the actual sizes of the populations by intersecting the given connection set with the Cartesian product of the neuron indices of the pre- and post-synaptic populations.
2. Connect the neurons. The weight, delay and any other synaptic parameters are taken from the connection set, if supplied as value sets (see section 3), or from the parameterization of the synapse type otherwise.

During this study, we extended the NEST backend for PyNN with a new and specialized CSAConnector which passes the complete ConnectionGenerator object down to the simulator (by calling PyNEST's CGConnect function). The iteration over the connections can then take place at the C++ level in NEST using the connection generator interface (see section 4). This greatly reduces the overhead and thus improves the runtime and scalability of connection generation (see section 6).

## 6. Benchmarks

We ran a series of benchmarks in order to assess the performance of and compare two implementations of CSA (see section 3.3) and the two implementations of the CSAConnector for PyNN (see section 5). Grouped by the software layer in which the iteration of the connection generator happens (either in Python by PyNN or in C++ by NEST, see section 4) and the CSA implementation used (csa is the Python version, libcsa the C++ version), the following scenarios were measured:

- **Python, csa** used PyNN's original CSAConnector that is available for all backends in combination with the Python CSA implementation. The CSAConnector intersects the csa object with a mask representing the actual source and target nodes available and iterates the csa object entirely at the Python level. It connects the neurons by issuing ConvergentConnect() calls to PyNEST.
- **C++, csa** used the new CSAConnector for PyNN's NEST backend, which is available in the development version of PyNN, and the Python CSA implementation. The csa object is passed down to NEST through CGConnect(). The ConnectionGeneratorModule then iterates it at the C++ level by repeatedly calling its Python level iterator through the C++ connection generator interface.
- **Python, libcsa** used the generic CSAConnector in PyNN and the C++ CSA implementation libcsa. The CSAConnector iterates the libcsa object at the Python level, repeatedly calling its wrapped C++ iterator.
- **C++, libcsa** used the new CSAConnector for PyNN's NEST backend and the C++ CSA implementation libcsa. The libcsa object is passed down to NEST through CGConnect(). All iterations happen in C++ in the ConnectionGeneratorModule through the connection generator interface.

**Figure 4:**
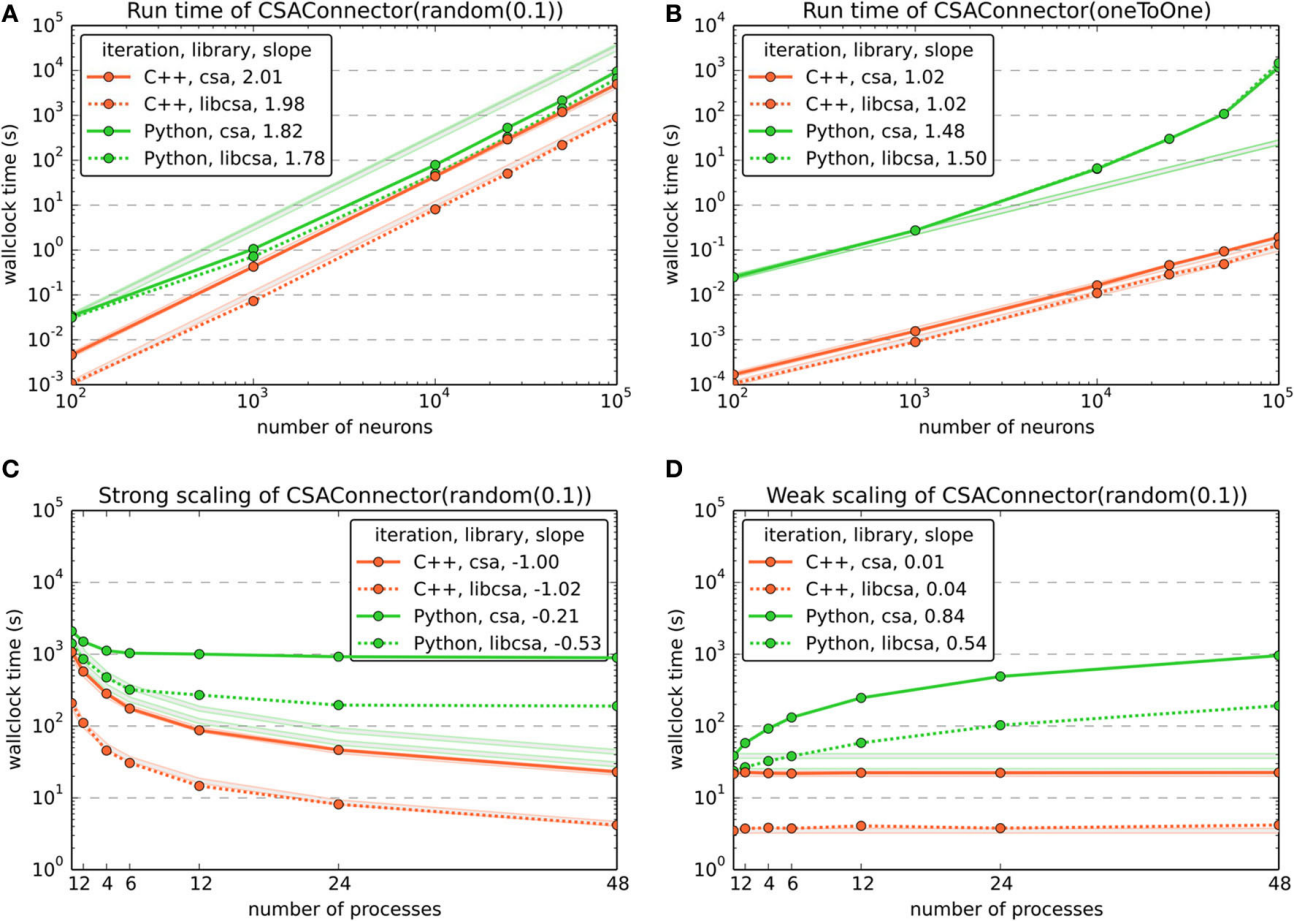
**Benchmark results for the use of CSA in NEST through PyNN, comparing the two CSAConnector implementations explained in section 5 and two of the CSA implementations mentioned in section 3**. Color and dash codes are given in the legends. Slope is the ratio of logarithms of the last and first data point shown. Pale lines denote the expected scaling. **(A)** Shows the run time for connecting a network using CSAs *random* mask with a probability of 0.1 for different numbers of neurons. This connector creates *O*(*n*^2^) connections for *n* neurons. The expected slope is thus 2. **(B)** Shows the same as in **(A)**, but using CSAs *oneToOne* mask, which creates *O*(*n*) connections for *n* neurons and has an expected slope of 1. **(C)** Shows a strong scaling experiment, wiring a network of 48,000 neurons using CSAs *random* mask with a probability of 0.1 and varying the number of MPI processes from 1 to 48. The expected slope is −1, meaning that the run time drops linearly with the number of processes. **(D)** Shows the results of a weak scaling experiment, increasing the number of connections by approx. 4.8 · 10^6^ per additional MPI process for 1 to 48 processes. The expected slope is 0, as the load increases linearly with the number of processes.

The population size benchmarks (Figures 4A,B) used one MPI process and varied the number of neurons from 10^2^ to 10^5^ with about one sample per order of magnitude. All tested implementations of CSA (csa, libcsa) scale excellently with slopes around 2 for the *random* mask, independent of the software layer (C++ or Python) at which the iteration was carried out. However, in Figure 4, the vertical offsets and the increasing slopes of the curves for the generic CSAConnector which iterates at the Python/PyNN level suggest that the current implementation of this connector together with calls through different software layers to setup individual connections adds a significant overhead to the process of connection generation.

To demonstrate the scalability of the implementations, we carried out both strong and weak scaling benchmarks, using CSA's *random* mask ρ(*p*) with a probability *p* = 0.1 (Figures 4C,D). For each of these, the number of MPI processes was varied from 1 to 48.

In the strong scaling benchmark (Figure 4C), the number of MPI processes was varied while keeping the total number of neurons in the network fixed at 48,000. This resulted in approx. 230 million connections being created. It is easily visible that iteration of the CSA object at the Python level in the current PyNN implementation of the original CSAConnector is detrimental to the scalability. In contrast, it is possible to obtain near perfect scaling if the iteration of the CSA object is carried out from the C++ layer. Using the C++ implementation of CSA, it is, however, possible to gain another order of magnitude compared to the Python version.

During the weak scaling benchmark (Figure 4C), the number of MPI processes was varied while the work load was increased by a fixed number of connections per additional process. The number of connections grows quadratically with the number of neurons when using CSA's *random* mask with a fixed probality. The number of neurons per process was thus increased with the square root of the desired number of connections. In the realm of natural numbers, this leads to a slight error, which was, however, acceptable, with values between −1.42‰ and +1.08‰.

### 6.1. Comparison to native interfaces

**Figure 5:**
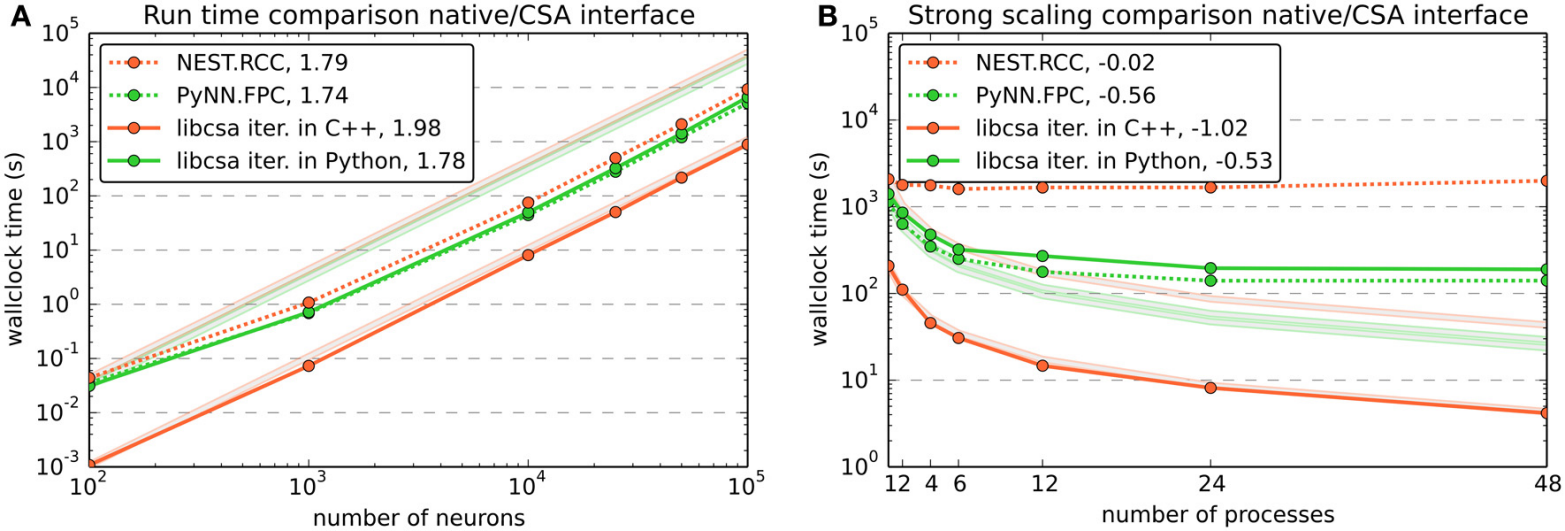
**Benchmark results comparing the use of CSA in NEST through PyNN and the native interfaces for random connectivity in PyNN and NEST**. Color and dash codes are given in the legends. Slope is the ratio of logarithms of the last and first data point shown. Pale lines denote the expected scaling. *PyNN.FCC* corresponds to PyNN's FixedProbabilityConnector, *nest.RCC* to NEST's function RandomConvergentConnect. **(A)** Shows the run time for connecting a network of different numbers of neurons. The number of connections scales with *O*(*n*^2^) for *n* neurons. The expected slope is thus 2. The data for libcsa is the same as shown in Figure 4A. **(B)** Shows a strong scaling experiment, wiring a network of 48,000 neurons using CSAs *random* mask with a probability of 0.1 and the corresponding native functions of PyNN and NEST. The experiment varies the number of MPI processes from 1 to 48. The expected slope is −1, meaning that the run time drops linearly with the number of processes. The data for libcsa is the same as shown in Figure 4C.

To compare the performance of the connection generator interface to more traditional ways of setting up connectivity, we also performed performance and scaling benchmarks of the native interfaces for random connectivity in PyNN and NEST. Figure 5 contrasts CSA's *random* mask with a probability of 0.1 with PyNN's FixedProbabilityConnector (FPC) and NEST's function RandomConvergentConnect (RCC).

Note that NEST does not provide a Bernoulli trial based connection scheme such as used by FPC in PyNN and the *random* mask in CSA (see section 3.2) and RCC was chosen because it is the function that most closely resembles such a method. The use of RCC results, however, in a bias toward the other methods over NEST, as it requires iterative calls to RCC from the Python level, which entail a certain overhead in SLI, PyNEST and the C++ implementation of RCC itself (data not shown).

The population size benchmark (Figure 5A) again used one MPI process and varied the number of neurons from 10^2^ to 10^5^ with about one sample per order of magnitude. It shows that all tested variants for setting up the connectivity scale equally well with slopes slightly below 2. The offset between NEST's RCC (written in C++, but called repeatedly from Python) to the CSA object iterated in C++ can be attributed to overhead in the additional software layers.

As in Figure 4C, the comparative strong scaling benchmark shown in Figure 5B varied the number of MPI processes while keeping the total number of neurons in the network fixed at 48,000, resulting in a total of approx. 230 million connections being created. The results are consistent with the data from Figure 4C in that scalability is destroyed if additional interpreted software layers are involved and that an iteration in C++ is favorable. The effect is even stronger for RCC, because its use to create a number of connections corresponding to that yielded by a Bernoulli trial scheme entails even more iterations through the additional software layers.

### 6.2. Benchmark environment

The machine used for benchmarking was equipped with 4 12-core AMD Operon 6174 (Magny Cours) processors, organized into 8 NUMA domains with 6 cores each. Other than the choice of the number of MPI processes in a NUMA-friendly way, no measures (like pinning of processes to cores, altering the affinity of threads to cores, etc.) were taken to avoid distortions of the results. Users are, however, free to benefit from such techniques to improve the performance of their simulations.

### 6.3. Software versions and benchmark scripts

All benchmarks were using csa revision 119 from http://svn.incf.org/svn/csa, libcsa from an internal git repository (git@wand.pdc.kth.se:libcsa.git, version 3ef2db519a), the development version of PyNN from https://github.com/NeuralEnsemble/PyNN/ (version db76c748cd) and NEST revision r10722 from the internal Subversion repository. The 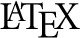 sources of this article, the benchmark scripts and all data we obtained are available from https://github.com/mdjurfeldt/pns2csa.

## 7. Discussion and outlook

We have developed and demonstrated a novel way to connect a simulator and external connectivity-generating software through the connection generator interface. It supports dynamic loading of connection generators without the need for recompilation of either simulator or generators. Benchmarks show good scaling when using connection-set algebra libraries from NEST and PyNN through the connection generator interface. The availability of this and associated interfaces lets users flexibly choose a connectivity-generating library independently of the simulator used, and thereby grants greater freedom for describing models.

The first version of the interface presented here was intentionally made simple, as a proof of concept, and there are several possible directions of development for its functionality. Currently, parameters are passed as doubles. This could be generalized to other data types. For parallel simulators, connection generators may need access to the MPI communicator in which case it needs to be passed through the connection generator interface. Instead of using a fixed iteration order, the order could be specified by the simulator. Iteration order could also be unspecified, giving opportunities for optimizing iteration on the part of the connectivity-generating library. In order to maintain backward compatibility as new revisions of the interface are developed, interface versioning could be introduced.

One temporary limitation in the current implementations of the NEST ConnectionGeneratorModule and the PyNN CSAConnector is that value sets for the connection generator can only be used if their arity is 0 (no parameters) or 2 (weight and delay). We are currently working on the infrastructure in NEST to support arbitrary arities, as it would be beneficial for many synapse models, to also set time constants, concentrations and other parameters based on rules in the connection generator.

At the moment, libneurosim only contains the connection generator interface and some auxiliary code. For the future, we are planning to turn this into a community based effort to share common interfaces and make it a generally useful library also for the developers of other simulators than PyNN and NEST.

The differences in run time and scalability between an iteration of the connection description in Python on the one hand and the CSA object iterated at the C++ level on the other suggests that a compact description of connectivity iterated solely at the level of the simulator indeed has a huge impact. NEST and other simulators can thus greatly benefit from the availability of compact descriptions such as enabled by the connection generator interface.

### Conflict of interest statement

The authors declare that the research was conducted in the absence of any commercial or financial relationships that could be construed as a potential conflict of interest.

## Footnotes

^1^CSA is an algebra in the sense of the word as used in “linear algebra” or “elementary algebra,” i.e., there exists a set of rules for how to manipulate connection-sets.

^2^In CSA notation, masks are marked with a bar.
